# Graph neural networks and belief rule base collaborative modeling for automated and interpretable fault diagnosis in proton exchange membrane fuel cells

**DOI:** 10.1371/journal.pone.0341884

**Published:** 2026-01-30

**Authors:** Yao Zhao, Ting Wang, Xin Wang

**Affiliations:** 1 School of Management, Guizhou University, Guiyang, China; 2 Guizhou Provincial Key Laboratory of Internet plus Intelligent Manufacturing, Guiyang, China; 3 School of Economics and Management, Guiyang University, Guiyang, China; Northwestern Polytechnical University, CHINA

## Abstract

Proton exchange membrane fuel cells (PEMFC) are critical for clean energy conversion, but their reliability is severely compromised by complex faults, creating a pressing need for accurate and interpretable diagnostic methods. While the Belief Rule Base (BRB) provides a transparent reasoning framework, its practical deployment faces two fundamental challenges: the “combinatorial explosion” of rules with increasing system complexity, and a heavy reliance on domain experts to provide precise quantitative parameters. To address these issues, this paper proposes a novel GNN-BRB framework that synergistically integrates Graph Neural Networks (GNN) with BRB. Our solution introduces two key innovations: an exponential ordered weighting operator to systematically convert qualitative expert rankings into quantitative confidence parameters, and a GNN-based mechanism that models the BRB rule base as a graph to automatically generate initial parameters through information propagation among semantically related rules. Experimental results on a real-world PEMFC fault diagnosis case demonstrate that the proposed method significantly reduces dependency on manual expert input while achieving superior diagnostic performance. Ablation studies further validate the contribution of each model component. This work establishes a new paradigm for developing automated, highly accurate, and interpretable fault diagnosis systems for complex engineering applications.

## 1. Introduction

Proton exchange membrane fuel cells (PEMFC) is an advanced technology for clean energy conversion, characterized by high efficiency, low emissions, and modular design [1]. Their application spans critical domains such as transportation, stationary power generation, portable electronics and electrochemical energy storage systems [2]. However, the operational reliability and durability of PEMFC are persistently challenged by complex fault conditions, including flooding, membrane drying, and gas starvation [3]. These faults arise from the intricate coupling of multi-physical processes (electrochemical, thermal, and fluid dynamic) within the cell, leading to performance degradation and potential irreversible damage if not diagnosed promptly. Consequently, the development of advanced fault diagnosis methodologies is paramount to ensuring system safety, extending service life, and facilitating the widespread commercialization of PEMFC technology [4–5].

Existing fault diagnosis approaches for PEMFC are generally categorized into model-based, data-driven, and knowledge-based methods. Model-based techniques use mathematical representations for interpretable diagnostics; however, their accuracy is limited by the difficulty of creating high-fidelity models for complex PEMFC dynamics [6–7]. Data-driven methods excel at automatic feature extraction; however, their performance is limited by data scarcity and their black-box nature, which hinders deployment in safety-critical scenarios [8]. In contrast, knowledge-based methods, such as expert systems, effectively utilize domain expertise and historical records, demonstrating particular robustness in scenarios with scarce data [9]. The Belief Rule Base (BRB) stands out as a powerful semi-quantitative modeling technique within this category, capable of integrating expert knowledge with quantitative data through IF-THEN rules embedded with confidence degrees [10]. This structure provides a transparent reasoning process, making BRB a compelling choice for PEMFC fault diagnosis.

Despite its strengths, the practical application of BRB in complex PEMFC systems faces two significant challenges. First, the construction of the rule base traditionally depends on manual parameter setting by domain experts. As the number of system monitoring variables increases, the number of rules grows exponentially, leading to a “combinatorial explosion” that makes manual configuration infeasible [11]. Second, experts often find it more intuitive to provide qualitative rankings (e.g., “symptom A is more indicative of fault X than symptom B”) rather than precise quantitative confidence values [12–13]. Translating such qualitative preferences accurately into the initial numerical parameters of a BRB remains a non-trivial and under-explored problem. While previous research has focused on optimizing BRB structure or reducing parameters [14–15], few studies address the automated generation of initial parameters by exploiting the potential correlations among rules or effectively converting qualitative rankings.

To address these critical gaps, this paper proposes a novel GNN-BRB framework that integrates Graph Neural Network (GNN) with Belief Rule Bases (BRB) for interpretable and automated fault diagnosis in PEMFC. The synergy of the two methods stems from the inherent graph structure of the BRB rule base, where rules act as nodes and their semantic similarities serve as edges. This structure allows the inherent interdependencies among rule attributes to be formalized, enabling the GNN to learn the underlying relational logic. Consequently, this approach facilitates large-scale, automatic rule generation and parameter optimization, thereby effectively mitigating the combinatorial explosion problem. Within the GNN-BRB architecture, the GNN component automates the knowledge engineering process, while the BRB component ensures the model’s overall transparency and interpretability. Specifically, this study makes the following key contributions:

(1) We introduce an exponential ordered weighting operator to systematically convert expert-provided qualitative preference rankings into quantitative BRB confidence parameters.
(2) We develop a GNN-based method that models the BRB rule base as a graph based on attribute similarity. This allows the GNN to propagate information and automatically generate initial parameters for large-scale BRB systems, drastically reducing the reliance on extensive expert input.
(3) We validate the effectiveness and superiority of the proposed GNN-BRB framework through a comprehensive case study on PEMFC fault diagnosis, demonstrating its high accuracy, robustness, and interpretability.

The rest of the paper is organized as follows: Section 2 briefly reviews the relevant research foundations and analyzes neglected issues in current research. Section 3 describes the components and workflow of GNN-BRB in detail. Section 4 uses a PEMFC fault diagnosis case to verify the effectiveness of the proposed method. This paper is concluded in Section 5.

## 2. Literature review

This section provides a comprehensive overview of existing research, divided into two subsections: first, focusing on PEMFC fault diagnosis methods, and then examining BRB-based approaches.

### 2.1. PEMFC fault diagnosis methods

Research on fault diagnosis for PEMFC systems primarily falls into three categories: model-based, data-driven, and knowledge-based methods. Among these, diagnosing transient faults (e.g., reactant supply, water management, and thermal management faults) is a critical focus due to their reversible nature and high incidence [4–16].

Model-based methods establish dynamic models of fuel cells to identify potential internal faults and generate diagnostic results by analyzing real-time monitoring data from sensors [7–17]. Data-driven methods rely on machine learning algorithms such as Bayesian networks [18], support vector machines (SVM) [19], or deep learning algorithms like long short-term memory (LSTM) [20] and convolutional neural networks (CNN) [21] to directly analyze data. These methods offer fast response and high accuracy, but require complex dynamic models and suffer from poor interpretability due to their inherent black-box nature, which limits their practical applications [22]. In contrast, knowledge-based methods leverage domain expertise effectively, demonstrating unique advantages in small-sample scenarios.

### 2.2. BRB-Based fault diagnosis methods

As a knowledge-based method, the BRB integrates expert systems with data-driven modeling, expressing qualitative knowledge through belief rules and combining it with quantitative data via reasoning and optimization [23]. It offers three unique advantages in fault diagnosis: knowledge fusion capability [24], white-box transparency [25], and low data dependency [26–27]. These characteristics make it particularly suitable for high-reliability applications where safety and interpretability are paramount, such as aerospace relay monitoring [28], marine engine wear analysis [29], and oil pipeline diagnostics [30]. Recent research has extended its application to complex health evaluations of wireless sensor networks [11] and inertial navigation systems [31].

Notwithstanding these strengths, a primary challenge for BRB application in complex systems is the combinatorial explosion of rules, where the rule base scale grows exponentially with the number of antecedent attributes and their reference levels [28]. To address this problem, researchers have proposed several optimization and structural strategies, which can be categorized into three main streams:

1. Dimension Reduction and Feature Selection: Traditional methods utilize Principal Component Analysis (PCA) to extract key features [32–33]. More recently, ensemble feature selection methods like DKH-EFS [34] and XGBoost-based techniques [35] have been introduced to select the most significant indicators while maintaining the physical meaning of attributes.
2. Structural Variants: To simplify rule configuration, the disjunctive BRB (DBRB) utilizes “OR” connectives to significantly reduce the rule count [31]. Other variants include the single-attribute belief rule base (ABRB) [36] and the ensemble belief rule base (EBRB) [37], the latter of which synchronizes the number of rules with the number of training samples to enhance modeling efficiency.
3. Hierarchical and Network Modeling: By increasing model depth to reduce layer width, hierarchical BRB (HBRB) [35] and multilayer BRB (ML-BRB) [38] effectively mitigate the explosion problem. Furthermore, the development of Belief Rule Networks (BRN) incorporates micro-belief rule structures and self-sampling criteria to handle high-dimensional inputs without information loss.

Despite progress in optimizing BRB structure and rule reduction, a foundational problem remains: the inefficient and subjective process of initial parameter setting, which relies heavily on manual expert input. It is non-standardized, inefficient, and prone to cognitive bias. In summary, while extant research has made progress in optimizing BRB structure and rule reduction, two critical gaps persist: the systematic conversion of qualitative rankings into quantitative BRB parameters, and the automated generation of initial parameters for large-scale BRB by leveraging inter-rule correlations. To bridge these gaps, this paper proposes the novel GNN-BRB collaborative modeling approach.

## 3. Methods

The proposed GNN-BRB model is detailed in this section, starting with foundational concepts, followed by model description, parameter generation, and reasoning stages.

### 3.1. Preliminary knowledge

The BRB is an expert knowledge-based reasoning system that effectively handles various uncertainties. Its core consists of multiple belief rules. A typical rule R_*k*_ in BRB can be formally expressed as follows:


$$R_{k}:If(x_{1}\;is\;A_{1}^{k})\wedge (x_{2}\;is\;A_{2}^{k})\wedge \cdots \wedge (x_{M_{k}}\;is\;A_{M_{k}}^{k}),$$

$$Then\;y\;\;is\{(D_{1},\beta_{1,k}),(D_{2},\beta_{2,k})\ldots (D_{N},\beta_{N,k})\}$$ (1)

Where the reference value of the i antecedent attribute is $A_{i}^{k}$(i = 1,2,…, M_*k*_), M_*k*_ denotes the number of antecedent attributes in the *k*-th rule, and $x_{1},x_{2},...,x_{M_{k}}$ are the input of the *k*-th rule. When it meets the reference value $A_{i}^{k}$(i = 1,2,…,*M*_*k*_), the output of the rule (called the consequent attribute) represents the *N* possible states of the system $D_{N}$ (N = 1,2,…,*M*_*k*_), with each state $D_{i}$ having an initial confidence degree $\beta_{i,k}$($\sum {\mathrm{\backslash nolimits}}_{i=1}^{N}\beta_{i,k}$ ≤1). Further, for different rules and antecedent properties, define $\theta_{k}$ as the weight of the *k*-th rule, and $\delta_{i}$ as the weight of the *i*-th antecedent attribute in the *k*-th rule.

In BRB rule construction, both the reference values of antecedent attributes and the initial confidence degrees affect the final decision outcomes of the rule base. When dividing antecedent attributes, all attributes that influence fault diagnosis must be considered, and each attribute’s reference levels must be categorized further. As the diagnosis target becomes more complex, the number of antecedent attributes or reference levels increases, which leads to exponential growth in BRB rules and potential combinatorial explosion. If initial confidence degrees $\beta_{i,k}$ rely on expert setting entirely, the accuracy and efficiency of manual annotation for large-scale rule bases are evidently challenging to guarantee.

### 3.2. Model description

**Fig 1:**
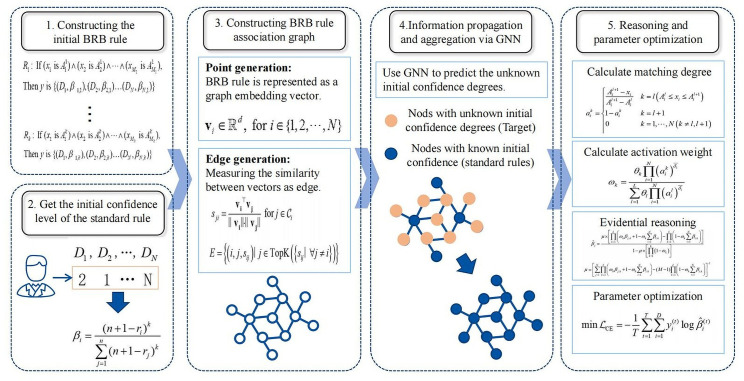
The process of the GNN-BRB model.

To address the aforementioned issues, this paper proposes a GNN-BRB model based on graph neural networks for learning expert knowledge and automatically generating BRB parameters. First, experts provide ranking values for a few typical fault states based on their knowledge. These values are then converted into probability values and used as node labels. Next, each BRB rule is represented as a node on a graph, and the associations between rules are modeled as edges. GNNs then aggregate and propagate information on the graph to predict labels for the remaining nodes. This process allows for the automatic filling of initial confidence degrees for large-scale BRB models with minimal expert input. The initial BRB model is then optimized using evidential reasoning and parameter optimization to obtain the final diagnostic results. As shown in Fig 1, the GNN-BRB model consists of two stages: BRB parameter generation and BRB reasoning with parameter optimization. These stages are detailed in Sections 3.3 and 3.4, respectively.

### 3.3. BRB parameter generation stage

#### 3.3.1. *Obtaining initial confidence degrees for standard rules.*

When constructing the initial BRB rules, some rules with typical causal relationships are selected as standard rules based on expert and prior knowledge. Experts rank the consequent attributes of these standard rules. For the *k*-th standard rule, if the input matches the antecedent attributes $A_{i}^{k}$, and the expert believes the likelihood of system states $D_{i}$ occurring is greater than that of states $D_{j}$, then $r_{i}$ <$r_{j}$ ($r_{i}$ indicating the ranking value of system state $D_{i}$ occurrence likelihood, where $r_{i},r_{j}\in 1\sim n$ and are integers). After ranking, we refer to the concept of the ordered weighted operators (OWA) [39] and design an exponentially ordered weighted transformation method that maps the ranking values to a probability distribution, where each option’s weight has an exponential relationship with its rank:


$$p_{i}=\frac{{\left(n+1-r_{i}\right)}^{k}}{\sum_{j=1}^{n}{\left(n+1-r_{j}\right)}^{k}}$$ (2)

Here, $r_{i}$ is the fault priority ranking provided by domain experts, k is a tuning parameter enhancing the distinction of ranking results, and $p_{i}$ is the initial confidence degree corresponding to the state $D_{i}$ after transformation.

#### 3.3.2. *Constructing the BRB rule association graph model.*

In a BRB rule base for complex system fault diagnosis, although each BRB rule represents different attributes of the diagnostic object, the rules may be associated due to internal system linkages. These associations are crucial prior knowledge in fault diagnosis decision-making. To utilize this prior knowledge effectively, each BRB rule is abstracted as a node on a graph, and a feature vector representing its characteristics is introduced. The associations between BRB rules are established based on the similarity of these feature vectors.

First, the feature vector ${𝐕}_{i}$ for the *i*-th BRB rule is defined as:


$${𝐕}_{i}\in \mathbb{R},fori\in \{1,2,\cdots ,N\}$$ (3)

For the *i*-th rule with P antecedent attributes, the possible value set for the *m*-th antecedent attribute $A_{m}$ is $S_{m}=\{v_{m1},v_{m2},...,v_{mk_{m}}\}$, where $k_{m}$ denotes the number of discrete values for the attribute. For the *m*-th attribute value $a_{im}$ of the *i*-th rule, a one-hot encoding vector ${𝐞}_{im}\in {\left\{0,1\right\}}^{km}$ is generated using a segment-based one-hot encoding method:


$${𝐞}_{im}[j]=\{\begin{array}{cc} {}*20l1, & when\;a_{im}=v_{mj}\\ 0, & others \end{array}$$ (4)

The global embedding vector $V_{i}$ is formed by concatenating the one-hot encodings of the P attributes:


$${𝐕}_{i}={𝐞}_{i1}⊕{𝐞}_{i2}⊕\cdots ⊕{𝐞}_{iP}\in \{0,1{\}}^{d}$$ (5)

Where ⊕ represents vector concatenation. Based on this, the edges of the graph are constructed by measuring the similarity between vectors as Equation (6). For vector $V_{i}$, the edges E are built by retaining the top *K* neighbors with the highest similarity, as Equations (7). The BRB rule association graph, denoted as $G_{s}$, is constructed accordingly：


$$s_{ji}=\frac{v_{i}{}^{⊤}v_{j}}{\|v_{i}\|\cdot \|v_{j}\|}\;for\;j\in {𝒞}_{i}$$ (6)

$$E=\left\{\left(i,j,s_{ij}\right)|\;j\in TopK\left(\left\{s_{ij}|\forall j\neq i\right\}\right)\right\}$$ (7)

#### 3.3.3. *Information propagation and aggregation via graph neural networks.*

On the constructed BRB rule association graph $G_{s}$, information propagation and aggregation are performed using GNN to obtain initial confidence degrees for other rules using the initial confidence degrees of the standard rules.

Consider an attribute graph $G_{s}=(𝒱,ℰ,A)$, where 𝒱 denotes the set of nodes, with each node’s feature being a *d*-dimensional vector $A\in {\mathbb{R}}^{N\times d}$, and the edge weights ℰ are calculated based on the Equation (6) in Section 3.2.2.

For a specific node K, representing the *k*-th BRB rule, its output corresponds to N possible system states *D*_*N*_ (N = 1,2,…,*M*_*k*_), with each state *D*_*i*_ having an initial confidence degree $\beta_{i,k}$($\beta_{i,k}$≤1). The label vector for node K is denoted as $L_{k}\in {\mathbb{R}}^{N},fork\in \{1,2,\cdots ,N\}$, with *N* dimensions, and the value of dimension i is $\beta_{i,k}$. Initially, only a few standard rule nodes have known label values. The GNN-BRB model aims to learn and generate a label matrix $L\in [0,1{]}^{N\times d}$ such that each dimension of L is close to the label values of neighboring nodes.

Based on the spectral convolution GNN model [40], a hierarchical message-passing architecture is designed. The hidden state $h_{i}^{\left(l\right)}$ update for node i at the l -th layer is:


$$h_{i}^{\left(l+1\right)}=\phi \left(\sum_{j\in 𝒩(i)}\frac{E_{ij}}{\sqrt{{\tilde{D}}_{ii}{\tilde{D}}_{jj}}}W^{\left(l\right)}h_{j}^{\left(l\right)}\right)$$ (8)

𝒩(i) is the set of neighbors for node i, $\overset{\sim }{D}\;{}_{ii}$ and $\overset{\sim }{D}{}_{jj}$ are the node i and j normalized degree matrix, $W^{\left(l\right)}$ are learnable parameters, and ReLU is the activation function ϕ(·). By stacking GNN layers, the model gradually aggregates neighborhood information. The label projection layer maps the final node embedding $H^{\left(L\right)}\in {\mathbb{R}}^{N\times d_{h}}$ to the constrained label space:


$$L=softmax\left(h^{\left(L\right)}\Theta_{out}+b_{out}\right)$$ (9)

Where $\Theta_{out}\in {\mathbb{R}}^{d_{h}\times d}$ is the dimensionality adaptation projection matrix, $b_{out}$ is the bias term, and softmax is the activation function.

#### 3.3.4. *Loss function.*

We employ mean squared error (MSE) as loss function in Equation (10), where *N* is the number of rules, $l_{i,k}$ is the true confidence degree for state in the *k*-th rule, and $\overset{\wedge }{\underset{i,k}{l}}$ is the model-predicted confidence degree for state in the *k*-th rule. By minimizing the difference between the predicted label $\overset{\wedge }{\underset{i,k}{l}}$ and $l_{i,k}$, it effectively captures the spatial distribution characteristics of probability vectors. The loss function is optimized using the Adam optimizer.


$${ℒ}_{MSE}=\frac{\text{1}}{N}\sum_{k=1}^{N}\sum_{i=1}^{n}{\left(l_{i,k}-{\overset{\wedge }{l}}_{i,k}\right)}^{2}$$ (10)

### 3.4. *Reasoning and parameter optimization stage*

#### 3.4.1. *BRB rule reasoning based on evidential reasoning algorithm.*

After obtaining the initial BRB rules, evidential reasoning algorithms are used for rule combination [41]. First, the matching degree of antecedent attributes is calculated, which determines the extent to which the input matches a rule’s antecedent attributes. The matching degree for the *k*-th rule’s antecedent attributes is calculated as follows:


$$\alpha_{i}^{k}=\{\begin{array}{cc} {}*20l\frac{A_{i}^{l+1}-x_{i}}{A_{i}^{l+1}-A_{i}^{l}}, & k=l\;\;\left(A_{i}^{l}\leq x_{i}\leq A_{i}^{l+1}\right)\\ 1-\alpha_{i}^{k}, & k=l+1\\ 0, & k=1,\ldots ,N\;\;\left(k\neq l,l+1\right) \end{array}$$ (11)

Where $a_{i}^{k}$ represents the matching degree of the *i*-th antecedent attribute in the *k*-th rule, $A_{i}^{l}$ and $A_{i}^{l+1}$ are the reference values of the *i*-th antecedent attribute in adjacent rules.

In the BRB model, input data activates rules based on matching differences. The activation weight for the *k*-th rule is calculated as Equation (12):


$$\omega_{k}=\frac{\theta_{k}\prod_{i=1}^{N}{\left(a_{i}^{k}\right)}^{\overset{―}{\delta_{i}}}}{\sum_{l=1}^{L}\theta_{l}\prod_{i=1}^{N}{\left(a_{i}^{l}\right)}^{\overset{―}{\delta_{i}}}}$$ (12)

Where $\omega_{k}$ is the activation weight of the *k*-th rule, $\theta_{k}$ is the rule weight, δ is the attribute weight, and $a_{i}^{k}$ is the matching degree of the input relative to the *i*-th attribute in the *k*-th rule. Finally, the evidential reasoning algorithm combines all rules in the BRB to obtain the final output:


$$S(x)=\left\{\left(D_{j},{\hat{\beta }}_{j}\right),\;j=1,2,\cdots ,N\right\}$$ (13)

Where ${\hat{\beta }}_{j}$ is the confidence degree relative to the evaluation result $D_{j}$, with:


$${\hat{\beta }}_{j}=\frac{\mu \times \left[\prod_{k=1}^{L}\left(\omega_{k}\beta_{j,k}+1-\omega_{k}\sum_{i=1}^{N}\beta_{i,k}\right)-\prod_{k=1}^{L}\left(1-\omega_{k}\sum_{i=1}^{N}\beta_{i,k}\right)\right]}{1-\mu \times \left[\prod_{k=1}^{L}\left(1-\omega_{k}\right)\right]}$$ (14)

$$\mu ={\left[\sum_{j=1}^{N}\prod_{k=1}^{L}\left(\omega_{k}\beta_{j,k}+1-\omega_{k}\sum_{i=1}^{N}\beta_{i,k}\right)-(M-1)\prod_{k=1}^{L}\left(1-\omega_{k}\sum_{i=1}^{N}\beta_{i,k}\right)\right]}^{-1}$$ (15)

#### 3.4.2. *Parameter optimization.*

For the initial BRB rules generated by the graph neural network in section 3.3, it is necessary to train and adjust the parameters through actual fault diagnosis data in order to achieve the optimal performance of the model. The precision of the BRB model is represented by cross-entropy loss. The optimization objective is calculated as Equation (16), where T is the number of data points, and D is the fault classification:


$$\min{ℒ}_{CE}=-\frac{\text{1}}{T}\sum_{t=1}^{T}\sum_{i=1}^{D}y_{i}^{\left(t\right)}\log{\hat{\beta }}_{i}^{\left(t\right)}$$ (16)

The P-CMA-ES algorithm, an improved multi-objective optimization method based on covariance matrix adaptation evolution strategy, is used to optimize the BRB [42]. After optimization, the confidence degrees in the BRB rules are updated to construct a more accurate fault diagnosis model.

### 3.5. Ethical approval and consent

The study protocol was approved by the Institutional Review Board of Guizhou University, Guiyang 550025, China. Written informed consent was obtained from all individual participants included in the study.

## 4. Results

This section presents a comprehensive evaluation of the proposed GNN-BRB model for PEMFC fault diagnosis. We begin by describing the data preparation process. Subsequently, we detail the model’s performance, starting from its initial state after the GNN-based parameter generation, through its optimized state after training, and finally comparing it against established benchmarks. An extensive ablation study is then conducted to validate the contribution of key model components and parameters, followed by an analysis of interpretability and the impact of prior knowledge.

### 4.1. *Data preparation*

#### 4.1.1. *PEMFC system introduction.*

**Fig 2:**
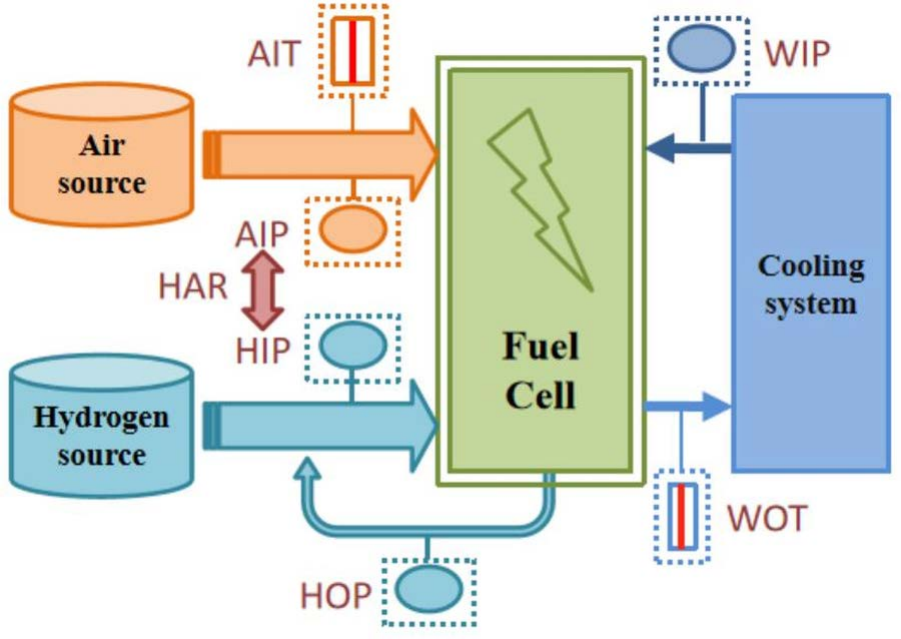
Components of the PEMFC used in this paper.

We analyze the fault diagnosis method proposed in this article using operating data from a 60 KW PEMFC. A PEMFC consists of stack, air system, hydrogen system, cooling liquid system, and control system. The system generates electricity through an electrochemical reaction of hydrogen and air, discharging water in the process. Fig 2 presents a simplified schematic diagram of the key components and monitoring points within the PEMFC system used in this study. The core of the system is the Fuel Cell Stack, where the electrochemical reaction occurs. The diagram illustrates the supply of reactants from the Air source and Hydrogen source. Critical operational parameters are monitored at the inlets and outlets: Air Inlet Pressure (AIP), Hydrogen Inlet Pressure (HIP), and Hydrogen Outlet Pressure (HOP). The pressure differential between the hydrogen and air supplies, known as the Hydrogen-Air pressure Residual (HAR), is also a key monitored variable. For the thermal management system, the Water Inlet Pressure (WIP) and Water Outlet Temperature (WOT) of the coolant are measured. The strategic placement of these sensors provides comprehensive real-time data on the system’s operational state, which serves as the antecedent attributes for the fault diagnosis model.

#### 4.1.2. *Data preparation.*

The experimental data comes from the long-term operational monitoring records of a PEMFC system. For each fault type, 100 data sets were collected, yielding a total of 600 data sets across six states (the normal state plus five fault states). The raw sampling interval of the sensors is 50ms; to suppress disturbances from data fluctuations and other anomalies, every ten raw samples were averaged into one data point, resulting in a continuous acquisition time of 50 s for a single fault. The data were randomly sampled and divided into training and testing sets: 80% (480 data points) for training and 20% (120 data points) for testing. The training set was used for the parameter optimization stage of the BRB model (Section 3.4.2), while the testing set, which includes all six states, was held out for the final performance evaluation. The specific data label divisions are shown in Table 1.

**Table 1:** PEMFC fault type and label.

Data number	1-100	101-200	201-300	301-400	401-500	501-600
Fault type	Normal	Flooding	Water shortage	Air starvation	Hydrogen starvation	Temperature abnormalities
Label	0	1	2	3	4	5

After consulting with experts, the key parameters of PEMFC operation were selected as the antecedent attributes in the BRB. These parameters include hydrogen inlet pressure (HIP), hydrogen outlet pressure (HOP), air inlet pressure (AIP), hydrogen air pressure residual (HAR), air inlet temperature (AIT), water outlet temperature (WOT), and water inlet pressure (WIP). Based on expert knowledge, three reference values were assigned to each antecedent attribute: Low (L), Normal (N), and High (H). This resulted in a total of 2187 BRB rules. The measured positions of each antecedent attribute are marked in Fig 2, and the division of the reference values for each antecedent attribute is shown in Table 2.

**Table 2:** Reference value division of each antecedent attributes.

Antecedent attributes	low (L)	normal (N)	high (H)
Hydrogen Inlet Pressure (HIP)	<1150 kPa	1150 kPa ~ 1500 kPa	>1500 kPa
Hydrogen Outlet Pressure (HOP)	<150 kPa	150 kPa ~ 270 kPa	>270 kPa
Air Inlet Pressure (AIP)	<100 kPa	100 kPa ~ 260 kPa	>260 kPa
Hydrogen Air pressure Residual (HAR)	<0 kPa	0 kPa ~ 20 kPa	>20 kPa
Air Inlet Temperature (AIT)	<−40 °C	−40 °C ~ 75 °C	>75 °C
Water Outlet Temperature (WOT)	<40 °C	40 °C ~ 80 °C	>80 °C
Water Inlet Pressure (WIP)	<150 kPa	150 kPa ~ 230 kPa	>230 kPa

### 4.2. *Experimental results*

#### 4.2.1. *Model performance evaluation indicators.*

This article uses the widely used indicators of recall, precision, and F1 score to evaluate the diagnostic performance of the model. Recall reflects the model’s ability to identify all actual fault samples; it represents the proportion of fault samples correctly identified by the model among all actual fault samples. Precision reflects the accuracy of the model’s prediction of fault samples; that is, it represents the proportion of actual fault samples among all samples predicted as faults by the model. The F1 score is a comprehensive indicator that reflects both the accuracy and breadth of the diagnostic results simultaneously. The formulas for calculating the recall rate, accuracy rate, and F1 score are as follows:


$$Recall@k=\frac{n_{TP}^{k}}{n_{TP}^{k}+n_{FN}^{k}}$$ (17)

$$Precision@k=\frac{n_{TP}^{k}}{n_{TP}^{k}+n_{FP}^{k}}$$ (18)

$$F1@k=\frac{2*\mathrm{Pr}e@k*Re\mathrm{\backslash nolimits}c@k}{\mathrm{Pr}e@k+Re\mathrm{\backslash nolimits}c@k}$$ (19)

Among them, TP (true positive) represents the actual fault samples that the model successfully identified. FN (false negative) represents the actual fault samples that the model failed to recognize. FP (false positive) indicates that the model incorrectly predicted normal samples as faulty.

#### 4.2.2. *Fault diagnosis performance of the initial GNN-BRB model.*

Before generating the initial GNN-BRB parameters, experts analyzed the PEMFC fault characteristics and selected 60 standard rules for annotation. For each typical fault type, they selected 10 rules as standard rules. Following the common practice in existing literature [43−44], the initial values of the rule and antecedent attribute weights were set to 1. Subsequently, these weights are treated as parameters to be refined through data-driven optimization to reflect the actual system dynamics. Meanwhile, the initial confidence of the standard rules was determined by the experts based on their prior knowledge. The tuning parameter k of our proposed exponential OWA as defined in Eq. (2) was set to 3 based on preliminary experiments. The remaining rule parameters were generated using the method described in Section 3.3. The model in Section 3.3 uses a three-layer GCN architecture with a middle layer dimension of 256 and an output dimension of 6, which corresponds to the multi-label probability prediction task. The model is optimized using the Adam optimizer with a learning rate of 0.005 and L2 regularization of 5e-4.

**Fig 3:**
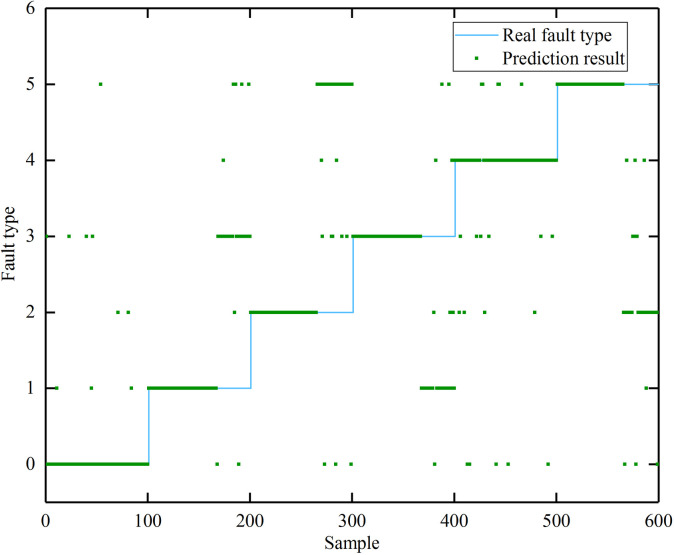
The initial GNN-BRB model fault diagnosis results.

Table 3 shows the initial parameter generation process by the GNN-BRB model. Due to the large number of rules, the table only shows some of them, with the colored parts provided by experts and the rest generated automatically by the model. Fig 3 shows the initial GNN-BRB diagnostic results, with an accuracy of 74%, a recall rate of 83%, and an F1 value of 0.77. In Fig 3, the blue line represents the actual fault type, and the green line represents the fault type predicted by the initial GNN-BRB model. It can be seen that the model has a certain diagnostic accuracy at this point, especially for the diagnosis of normal state (label 0).

**Table 3:** The initial parameter generation process by the GNN-BRB model.

No.	Rule weight	Attribute							Label					
		HIP	HOP	AIP	HAR	AIT	WOT	WIP	0	1	2	3	4	5
**Select a few standard rules and experts sort the attributes of the subsequent items**														
1	1	L	L	L	L	L	L	L	/	/	/	/	/	/
2	1	L	L	L	L	L	L	N	1	6	6	6	6	6
3	1	L	L	L	L	L	L	H	/	/	/	/	/	/
4	1	L	L	L	L	L	N	L	/	/	/	/	/	/
5	1	L	L	L	L	L	N	N	6	6	6	6	1	6
6	1	L	L	L	L	L	N	H	/	/	/	/	/	/
7	1	L	L	L	L	L	H	L	2	1	6	6	6	6
8	1	L	L	L	L	L	H	N	/	/	/	/	/	/
9	1	L	L	L	L	L	H	H	/	/	/	/	/	/
10	1	L	L	L	L	N	L	L	/	/	/	/	/	/
**......**														
**After sorting is completed, map the sorted values to a probability distribution**														
1	1	L	L	L	L	L	L	L	/	/	/	/	/	/
2	1	L	L	L	L	L	L	N	0.9774	0.0045	0.0045	0.0045	0.0045	0.0045
3	1	L	L	L	L	L	L	H	/	/	/	/	/	/
4	1	L	L	L	L	L	N	L	/	/	/	/	/	/
5	1	L	L	L	L	L	N	N	0.0045	0.0045	0.0045	0.0045	0.9774	0.0045
6	1	L	L	L	L	L	N	H	/	/	/	/	/	/
7	1	L	L	L	L	L	H	L	0.3623	0.6261	0.0029	0.0029	0.0029	0.0029
8	1	L	L	L	L	L	H	N	/	/	/	/	/	/
9	1	L	L	L	L	L	H	H	/	/	/	/	/	/
10	1	L	L	L	L	N	L	L	/	/	/	/	/	/
**......**														
**Using graph neural networks to obtain the initial confidence values of other rules**														
1	1	L	L	L	L	L	L	L	0.7556	0.0404	0.0072	0.1148	0.0518	0.0301
2	1	L	L	L	L	L	L	N	0.9774	0.0045	0.0045	0.0045	0.0045	0.0045
3	1	L	L	L	L	L	L	H	0.2094	0.3618	0.1072	0.1072	0.1072	0.1072
4	1	L	L	L	L	L	N	L	0.0489	0.0201	0.2538	0.0301	0.6287	0.0185
5	1	L	L	L	L	L	N	N	0.0045	0.0045	0.0045	0.0045	0.9774	0.0045
6	1	L	L	L	L	L	N	H	0.1307	0.0574	0.0493	0.0665	0.6737	0.0222
7	1	L	L	L	L	L	H	L	0.3623	0.6261	0.0029	0.0029	0.0029	0.0029
8	1	L	L	L	L	L	H	N	0.0502	0.0102	0.0418	0.0299	0.8603	0.0076
9	1	L	L	L	L	L	H	H	0.1085	0.2012	0.1888	0.1294	0.2867	0.0853
10	1	L	L	L	L	N	L	L	0.6106	0.0938	0.0807	0.0471	0.1383	0.0295
**......**														

#### 4.2.3. *Fault diagnosis performance of the optimized GNN-BRB model.*

**Fig 4:**
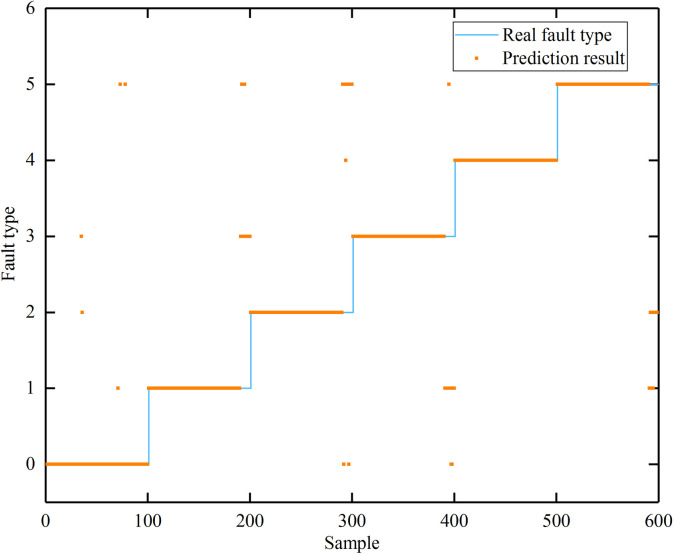
The optimized GNN-BRB model fault diagnosis results.

After generating the initial GNN-BRB parameters, optimize the parameters using the inference and optimization training method described in Section 3.4. The optimized GNN-BRB achieved an accuracy rate of 89%, a recall rate of 93%, and an F1 score of 0.91. All diagnostic results are shown in Fig 4. The blue line in Fig 4 represents the actual fault type, and the orange line represents the fault type predicted by the final BRB model. The model demonstrates good diagnostic accuracy for various fault types after parameter optimization. Notably, the model exhibits improved accuracy for distinguishing between easily confused fault types, such as flooding (label 1) and air starvation (label 3), as well as dehydration (label 2) and temperature control faults (label 5), compared to the initial model.

### 4.3. *Fault diagnosis performance comparison*

To validate the effectiveness of the proposed method, comparative experiments were conducted using two different parameter generation methods (BRB and three data-driven methods).

1) BRB methods: A comparative study was conducted using two methods to generate BRB parameters. The first method uses standard rule parameters provided by experts. Other parameters are randomly generated and recorded as partially given parameters BRB (PGP-BRB). The second method uses completely random parameters and is recorded as completely random parameters BRB (CRP-BRB). The parameter settings for these models were the same as in the example with 2,187 belief rules.
2) Data driven methods: Data driven methods are widely used in fault diagnosis, and we chosen three data-driven pattern classification methods, include Support Vector Machine (SVM) [45], Random Forest [46], and Artificial Neural Network (ANN) [47]. SVM constructs the optimal hyperplane in a high-dimensional feature space for classification, with the goal of maximizing the gap between different categories of data. Random forest is an ensemble learning method that constructs multiple decision trees and integrates their prediction results (voting or averaging). ANN is a feedforward neural network that includes an input layer, multiple hidden layers, and an output layer, by constructing a complex mapping relationship from input to output through the neural network and optimizing weights using backpropagation algorithm, ANN can achieve complex multi class prediction tasks.

Table 4 shows the diagnostic performance comparison between the GNN-BRB model proposed in this article and other comparative models. As can be seen, the GNN-BRB model significantly improves diagnostic performance compared with BRB models obtained by other parameter generation methods. This is manifested by the GNN-BRB model having significantly higher recall, accuracy, and F1 value than the PGP-BRB and CRP-BRB models. Compared to data-driven methods, the GNN-BRB model significantly outperforms SVM and RF and performs slightly better than the three-layer ANN model. However, the diagnostic results obtained by the GNN-BRB model are more interpretable than those obtained by the ANN model, making it particularly suitable for making fault diagnosis decisions.

**Table 4:** Comparison of the GNN-BRB model’s diagnostic performance with other methods.

Result	Methods						Promote
	PGP-BRB	CRP-BRB	SVM	RF	ANN	GNN-BRB	(%)
Precision	0.52	0.38	0.68	0.81	0.88	**0.89**	**1.1%↑**
Recall	0.62	0.45	0.72	0.85	0.91	**0.93**	**2.2%↑**
F1	0.57	0.41	0.70	0.83	0.89	**0.91**	**1.7%↑**

### 4.4. Model ablation analysis

To verify the effectiveness of the proposed method, we compared the impact of each model module on the results.

#### 4.4.1. *Impact of GNN information propagate and aggregation process on model performance.*

**Fig 5:**
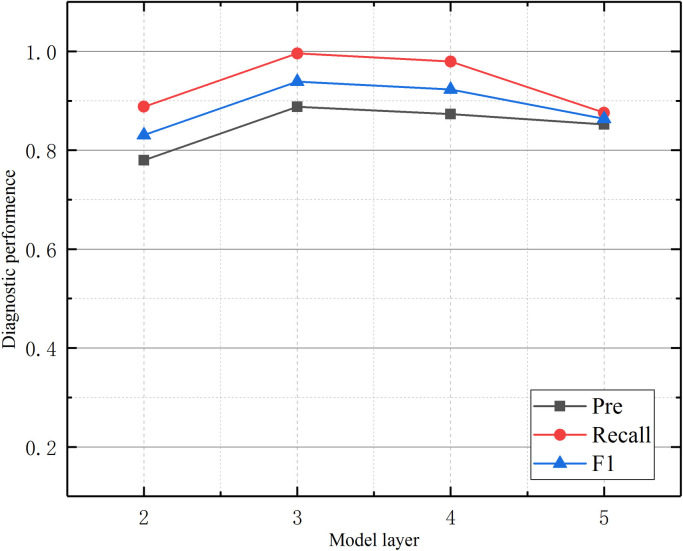
Influence of model layers on model diagnostic performance.

**Fig 6:**
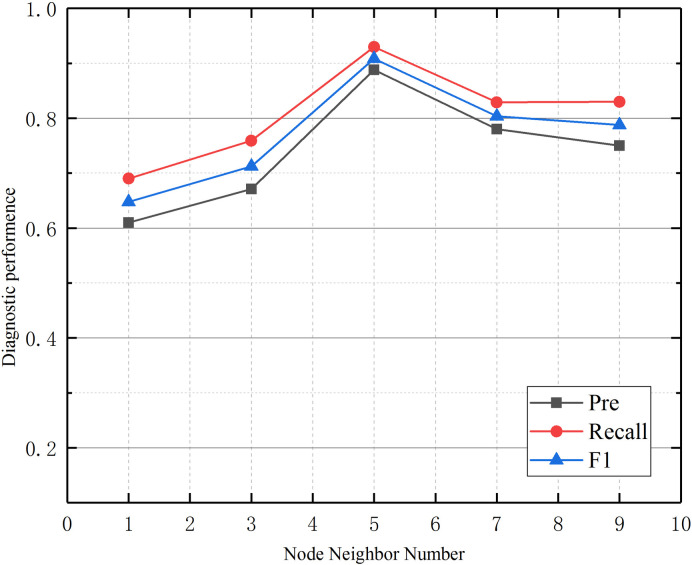
Influence of the number of node neighbors on model diagnostic performance.

Graph neural networks propagate and aggregate information through the topological relationships of graph structures. Their model performance is mainly affected by two key parameters. The number of network layers determines the distance of information propagation. The number of node neighbors (top_k) controls the scope of information aggregation. This section conducted comparative experiments on these two parameters: the number of model layers (2–5) and the number of neighbors (1–9). Each combination of parameters was subjected to ten repeated experiments, and the results are shown in Fig 5 and Fig 6.

As shown in Fig 5, the number of layers in a model significantly impacts its diagnostic performance. As the number of layers increases, the model’s diagnostic performance first increases and then decreases. The model’s performance is optimal with a three-layer network structure. This suggests that a three-layer network structure effectively balances representation learning and overfitting issues during information aggregation. In graph neural networks, the number of layers corresponds to the number of times node information propagates through the network. This study shows that, when information is propagated more than three times (i.e., when network layers are greater than three), node features gradually lose their discriminability due to excessive smoothing.

Fig 6 illustrates the effect of the number of node neighbors on diagnostic performance. As the number of node neighbors increases, we found that the model’s diagnostic performance first increases and then decreases. The model achieves its best performance when the number of node neighbors is 5, which indicates that the average number of rules with the highest correlation for a specific BRB rule is 5.

#### 4.4.2. *Ablation on confidence degree initialization methods.*

A pivotal contribution of our approach lies in translating expert qualitative rankings into quantitative BRB parameters via the exponential Ordered Weighted Averaging (OWA) operator. To rigorously validate the effectiveness of this specific design choice, we conducted an ablation study comparing different initialization methods for converting expert rankings into the initial confidence degrees of the standard rules. The GNN architecture (3 layers, top_k = 5) and the P-CMA-ES optimizer were kept identical across all experiments; only the method for generating the initial confidence labels for the standard rules was varied. The compared methods are:

**Exponential OWA (Proposed):** Our method, as defined in Eq. (2), which assigns weights that decay exponentially with the ranking order, sharply distinguishing the expert’s top preference.

**Linear OWA:** A baseline method using strictly linearly decreasing weights. The initial confidence degree corresponding to the state $D_{i}$ for the i -th rank is calculated as $p_{i}=\frac{2(N_{rank}+1-i)}{N_{rank}(N_{rank}+1)}$, where $N_{rank}$ is the number of entities being ranked. This creates a linear descent in weights from the highest to the lowest rank.

**One-Hot Encoding:** This method converts the expert’s top-ranked state into a one-hot vector, assigning it a confidence of 1.0 and all others 0. It represents an extreme case of absolute certainty based on the ranking.

The final performance of models using these different initialization schemes is compared in Table 5.

**Table 5:** Ablation study on confidence degree initialization methods.

Initialization Method	Precision	Recall	F1 Score
Linear OWA	0.75	0.81	0.78
One-Hot Encoding	0.83	0.62	0.71
Exponential OWA (Proposed)	0.89	0.93	0.91

The results confirm the superiority of the proposed Exponential OWA. While One-Hot Encoding is effective, its binary nature discards the nuance of expert ranking for non-primary states, limiting its recall performence. Linear OWA improves upon the uniform baseline but provides a less distinct signal than the exponential variant.

Our Exponential OWA optimally balances clarity and nuance. It strongly emphasizes the expert’s primary choice while preserving a structured probability mass for lower-ranked states, providing the GNN with the richest and most accurate supervisory signal for generating a high-performance initial BRB.

#### 4.4.3. *Model interpretability analysis.*

**Fig 7:**
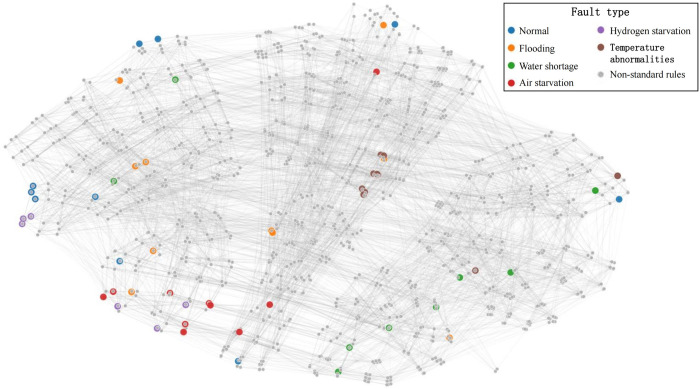
Distribution of standard rules in the initial state.

**Fig 8:**
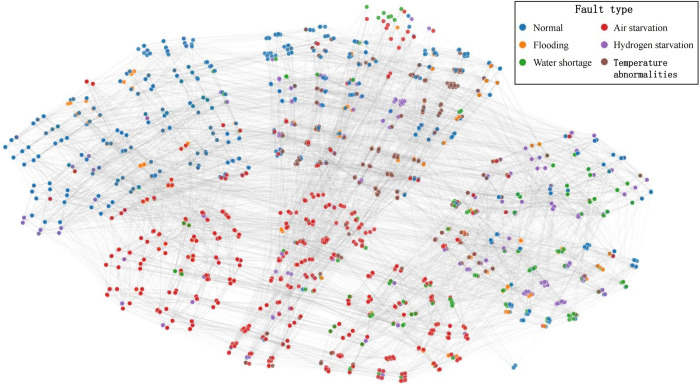
Distribution of final BRB rules generated by the GNN-BRB model.

To better demonstrate how the GNN-BRB model learns from expert-provided standard rules and generates remaining BRB rules, we using the t-SNE algorithm [48] to network visualized the information propagation and aggregation process in Section 3.3, as shown in Fig 7 and Fig 8. In these figures, each node represents a BRB rule, and the rule’s label is the one with the highest initial confidence among the system states output by that rule. Fig 7 shows each standard rule at the initial state, and Fig 8 shows the final BRB rules generated by the GNN-BRB model. As can be seen, each rule exhibits local clustering in the projected two-dimensional space, verifying that the learned feature representation effectively reflects the local correlations present in BRB rules.

#### 4.4.4. *Impact of prior knowledge on model performance.*

**Fig 9:**
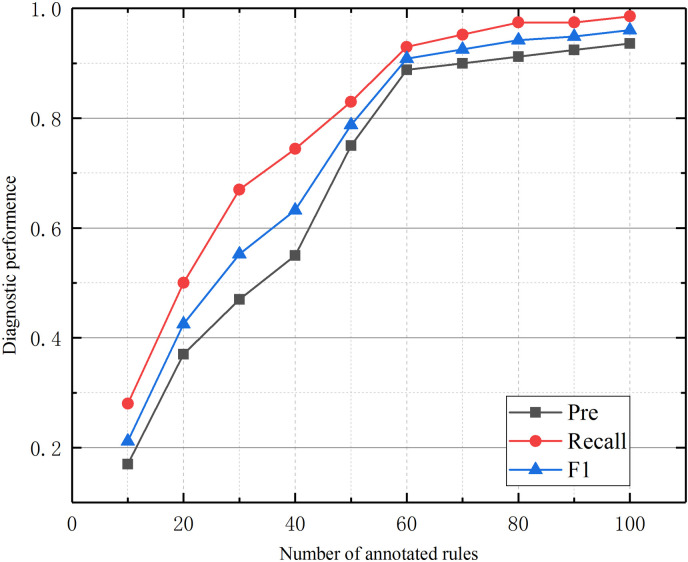
Impact of the number of expert annotation rules on model diagnostic performance.

Another research focus of this article is how to generate initial values for BRB models using incomplete expert prior knowledge. Therefore, in the data preparation stage of model training, experts annotate some typical rules. To verify how the number of expert annotation rules affects the performance of the model, we validated the diagnostic results of the model when the standard rules were validated at intervals of 10 within the range of 10–100, as shown in Fig 9.

It can be seen that as the number of annotation rules increases, the model’s diagnostic performance improves. In other words, the more prior knowledge that is injected into the system, the better the model performs. However, this growth is not linear but gradually slows down. This suggests that when the model has limited prior knowledge, adding a small amount can quickly improve its diagnostic performance. However, once the model has learned most of the prior knowledge, adding more does not significantly improve its performance. Considering that obtaining standard rules requires manual annotation by experts, these performance improvements are clearly not endless.

## 5. Conclusion

This paper proposes an innovative collaborative modeling approach for Graph Neural Network (GNN) and Belief Rule Base (BRB). First, the method converts expert qualitative ranking knowledge into BRB parameters by defining an exponentially weighted ordered weighting operator. Then, it exploits the correlation between BRB rules for graph modeling and uses GNN for graph learning. This two-stage process automatically generates a large-scale rule base with only a small amount of expert knowledge. The GNN-BRB model was validated using real fuel cell data and demonstrated excellent fault diagnosis performance. Ablation studies confirm that the number of GNN layers affects information propagation distance, and the number of adjacent layers affects information aggregation scope. The injection of prior knowledge exhibits a trend of diminishing marginal benefits. Compared to data-driven models, such as artificial neural networks (ANN), this method is interpretable while maintaining high accuracy, making it suitable for security-sensitive domains. This work not only provides a robust solution for PEMFC health management but also establishes a new paradigm for injecting and automating expert knowledge in interpretable AI models for complex engineering systems. Future research will explore dynamic knowledge update mechanisms to meet real-time diagnostic needs in time-varying scenarios.
